# Phasic left atrial strain to predict worsening of diastolic function: Results from the prospective Berlin Female Risk Evaluation follow-up trial

**DOI:** 10.3389/fcvm.2023.1070450

**Published:** 2023-02-20

**Authors:** Anna Brand, Elena Romero Dorta, Adrian Wolf, Daniela Blaschke-Waluga, Ute Seeland, Claudia Crayen, Sven Bischoff, Isabel Mattig, Henryk Dreger, Karl Stangl, Vera Regitz-Zagrosek, Ulf Landmesser, Fabian Knebel, Verena Stangl

**Affiliations:** ^1^Department of Cardiology and Angiology, Charité – Universitätsmedizin Berlin, Campus Mitte, Berlin, Germany; ^2^Department of Cardiology, Charité – Universitätsmedizin Berlin, Campus Benjamin Franklin, Berlin, Germany; ^3^DZHK (German Centre for Cardiovascular Research), Partner site Berlin, Berlin, Germany; ^4^Institute of Social Medicine, Epidemiology and Health Economics, Charité – Universitätsmedizin Berlin, Berlin, Germany; ^5^Department of Education and Psychology, Freie Universität Berlin, Berlin, Germany; ^6^Institute of Gender in Medicine, Charité – Universitätsmedizin Berlin, Berlin, Germany; ^7^University Hospital Zürich, University of Zürich, Zürich, Switzerland; ^8^Clinical Department of Cardiology, Internal Medicine II, Sana Klinikum Lichtenberg, Berlin, Germany

**Keywords:** left atrial strain, diastolic dysfunction, left atrium, LAVI, BEFRI

## Abstract

**Purpose:**

The predictive value of maximum left atrial volume index (LAVI), phasic left atrial strain (LAS) and other standard echocardiographic parameters assessing left ventricular (LV) diastolic function to discriminate a future worsening of diastolic function (DD) in patients at risk is unclear. We aimed to prospectively assess and compare the clinical impact of these parameters in a randomly selected study sample of the general urban female population.

**Methods and results:**

A comprehensive clinical and echocardiographic evaluation was performed in 256 participants of the Berlin Female Risk Evaluation (BEFRI) trial after a mean follow up time of 6.8 years. After an assessment of participants’ current DD status, the predictive impact of an impaired LAS on the course of DD was assessed and compared with LAVI and other DD parameters using receiver operating characteristic (ROC) curve and multivariate logistic regression analyses. Subjects with no DD (DD0) who showed a decline of diastolic function by the time of follow-up showed a reduced LA reservoir (LASr) and conduit strain (LAScd) compared to subjects who remained in the healthy range (LASr 28.0% ± 7.0 vs. 41.9% ± 8.5; LAScd −13.2% ± 5.1 vs. −25.4% ± 9.1; *p* < 0.001). With an area under the curve (AUC) of 0.88 (95%CI 0.82–0.94) and 0.84 (95%CI 0.79–0.89), LASr and LAScd exhibited the highest discriminative value in predicting worsening of diastolic function, whereas LAVI was only of limited prognostic value [AUC 0.63 (95%CI 0.54–0.73)]. In logistic regression analyses, LAS remained a significant predictor for a decline of diastolic function after controlling for clinical and standard echocardiographic DD parameters, indicating its incremental predictive value.

**Conclusion:**

The analysis of phasic LAS may be useful to predict worsening of LV diastolic function in DD0 patients at risk for a future DD development.

**GRAPHICAL ABSTRACT:**
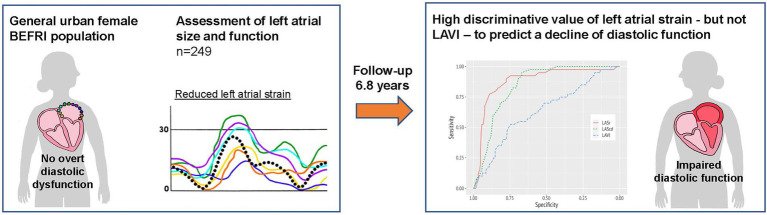

## Introduction

The importance of assessing the left atrium (LA) in evaluating left ventricular (LV) diastolic function is well established (1–3). The presence of early staged LV diastolic dysfunction (DD) has been related to worse outcome and higher risk for developing heart failure with preserved ejection fraction (HFpEF) (4, 5). Latest updated recommendations of the 2016 guidelines of the American Society of Echocardiography (ASE) and European Association of Cardiovascular Imaging (EACVI) propose a simplified algorithm that predominantly focuses on the detection of increased LV filling pressures (LVFP) including early diastolic transmitral flow velocity in relation to tissue velocity during early diastole (E/e′), e′, maximum LA volume index (LAVI), and estimated pulmonary artery systolic pressure (6). Although this approach provides an adequate diagnostic accuracy when compared to invasively measured LVFP (7, 8), the presence and severity of LV DD remains undetermined in many patients (9–11). In addition, DD parameters that offer predictive information are so far lacking. While the current algorithm proposed in the ASE/EACVI guidelines yields high specificity for the detection of clinically overt DD (6), LA deformation analysis using 2D speckle tracking echocardiography (2D STE) may add important diagnostic value due to its high sensitivity to reveal subtle myocardial alterations (4) and its ability to detect gradual decline in myocardial diastolic performance (3). An increased LAVI has been related to persistent chronic elevation of LV/LA pressures; however, as the LA size may take time to remodel, LAVI has been shown to be an insensitive parameter in the early phases of DD (3, 9–11). In contrast, functional changes appear at prompt stages of DD (4, 9) providing not only a diagnostic advantage but also being helpful for categorizing its severity (10, 11).

The standardization of LA strain (LAS) nomenclature and analysis by 2D STE as well as the definition of normal reference ranges have been recently published (12, 13) making it a tool ready to use for clinical practice. The predictive value of phasic LAS is well established for risk assessment and for guiding therapeutic strategies in the setting of atrial fibrillation (14, 15), cardiac amyloidosis (16), and in acute heart failure (17). There is emerging evidence that LASr could be of important prognostic value in HFpEF, as well (18, 19). However, prospective data are scarce and the predictive impact of LAS alterations on the development of DD is still unclear. In the face of the increasing clinical and economic burden of advanced DD, the implementation of echocardiographic parameters that are not limited to a reliable diagnostic accuracy, but also offer predictive information seems crucial to identify patients at risk for a future development of DD.

Accordingly, the aim of our longitudinal study was to prospectively assess the clinical value of phasic LAS alterations to predict a worsening of diastolic function over time in a randomly selected sample of study participants without clinically overt DD.

## Methods

### Study population and classification

The Berlin Female Risk Evaluation (BEFRI) trial comprised a randomly selected urban female population aged 25–74 years. A detailed description of the BEFRI design has been already published (20). In 2013 and 2014, 473 women participants received a comprehensive transthoracic echocardiography comprising the prospective evaluation of phasic LA function. Data regarding the echocardiographic measurements, focusing on LV DD, have been released in detail previously (4). The study was approved by the institutional ethics committee of Charité-Universitätsmedizin Berlin (EA/2085/19) and all participants gave informed written consent. A subsample from this initial study data is used here as baseline measurements.

We reinvited every woman who participated in the BEFRI echo study feasible for the analysis of LA structure and function for follow-up examinations. These took place between October 2019 and December 2020. Next to the assessment of demographical and clinical data, an extensive transthoracic echocardiography was performed with the focus on DD assessment as well as on LA and LV strain analysis. Sample characteristics at baseline and follow-up are presented in Table 1. Somatometric measurements and clinical data for the larger sample at baseline have been already described in detail (4, 20).

**Table 1:** Demographic and clinical characteristics.

	Total sample	Subgroups				*p*-value
		DD0 at baseline		DD1 at baseline		
		No progress	Progress	No progress	Progress	
Group size	249	186	34	23	6	
**Baseline**					
Age, years	51.6 ± 13.5	47.6 ± 12.5	62.6 ± 9.2	64.0 ± 6.9	66.5 ± 7.7	<0.001
BMI, kg/m^2^	24.5 ± 4.5	23.8 ± 4.5	26.0 ± 3.9	26.8 ± 4.0	28.4 ± 4.8	<0.001
Hypertension, *n* (%)	59 (24)	25 (13)	19 (56)	11 (48)	4 (67)	<0.001
Diabetes, *n* (%)	12 (5)	6 (3)	3 (9)	2 (9)	1 (17)	0.11
CAD, *n* (%)	2 (<1)	0	1 (3)	0	1 (17)	0.11
RR syst, mmHg	122.5 ± 14.6	120.7 ± 14.5	128.7 ± 13.3	124.2 ± 11.1	136.0 ± 7.0	<0.001
RR diast, mmHg	73.1 ± 9.9	72.3 ± 10.0	75.3 ± 10.5	74.4 ± 8.9	79.3 ± 4.8	0.045
Heart rate, bpm	70.7 ± 10.5	70.9 ± 10.9	70.2 ± 9.2	71.0 ± 10.0	67.7 ± 3.7	0.91
BNP, pg/ml	32.3 ± 26.3	27.5 ± 2.4	39.9 ± 25.2	51.3 ± 44.9	60.7 ± 42.2	<0.001
Creatinine, μmol/L	62.9 ± 9.8	63.0 ± 9.5	62.6 ± 11.0	62.6 ± 10.2	63.5 ± 11.8	0.90
**Follow-up**						
BMI, kg/m^2^	25.3 ± 5.0	24.7 ± 5.0	26.3 ± 4.3	27.6 ± 4.1	30.0 ± 6.1	0.001
Hypertension, *n* (%)	78 (31)	36 (19)	22 (67)	15 (65)	5 (83)	<0.001
Diabetes, *n* (%)	15 (6)	6 (3)	5 (15)	3 (13)	1 (17)	0.009
CAD, *n* (%)	3 (1)	0	1 (3)	1 (4)	1 (17)	0.009
RR syst, mmHg	125.9 ± 11.6	123.6 ± 11.0	131.7 ± 11.3	133.8 ± 9.5	137.5 ± 8.8	<0.001
RR diast, mmHg	72.3 ± 7.6	71.2 ± 7.5	75.5 ± 6.6	75.8 ± 7.1	77.5 ± 6.1	0.001
Heart rate, bpm	68.9 ± 10.6	69.0 ± 10.6	68.5 ± 10.5	68.6 ± 11.1	61.3 ± 7.1	0.52
NTproBNP, pg/ml	118.3 ± 117.5	91.0 ± 64.7	219.5 ± 226.7	158.6 ± 91.8	223.2 ± 169.2	<0.001

Values are *n* (%) or mean ± SD; DD0, normal diastolic function; DD1, impaired diastolic function; BMI, body mass index; CAD, coronary artery disease; RR, syst and diast, systolic and diastolic blood pressure.

### Standard echocardiography and strain imaging

A comprehensive transthoracic echocardiographic examination was performed using the same system applied for baseline examinations (Vivid E9 system, GE Vingmed, Horton, Norway, with an M5S 1.5- to 4.5-MHz transducer). The predefined echocardiographic study protocol can be inspected in the Supplementary material. Routine echocardiographic and Doppler data were obtained in accordance with the current ASE guidelines (6, 21). Standard parameters to assess diastolic function included LAVI; diastolic transmitral inflow velocities derived from pulsed wave-Doppler signal as well as the deceleration time; the septal, lateral, or average early diastolic mitral annular velocity (e′) assessed by pulsed-wave tissue Doppler; and E/e′ ratio. The RV-RA pressure difference was estimated from the maximum transvalvular velocity of the tricuspid regurgitation during systole.

**Figure 1:**
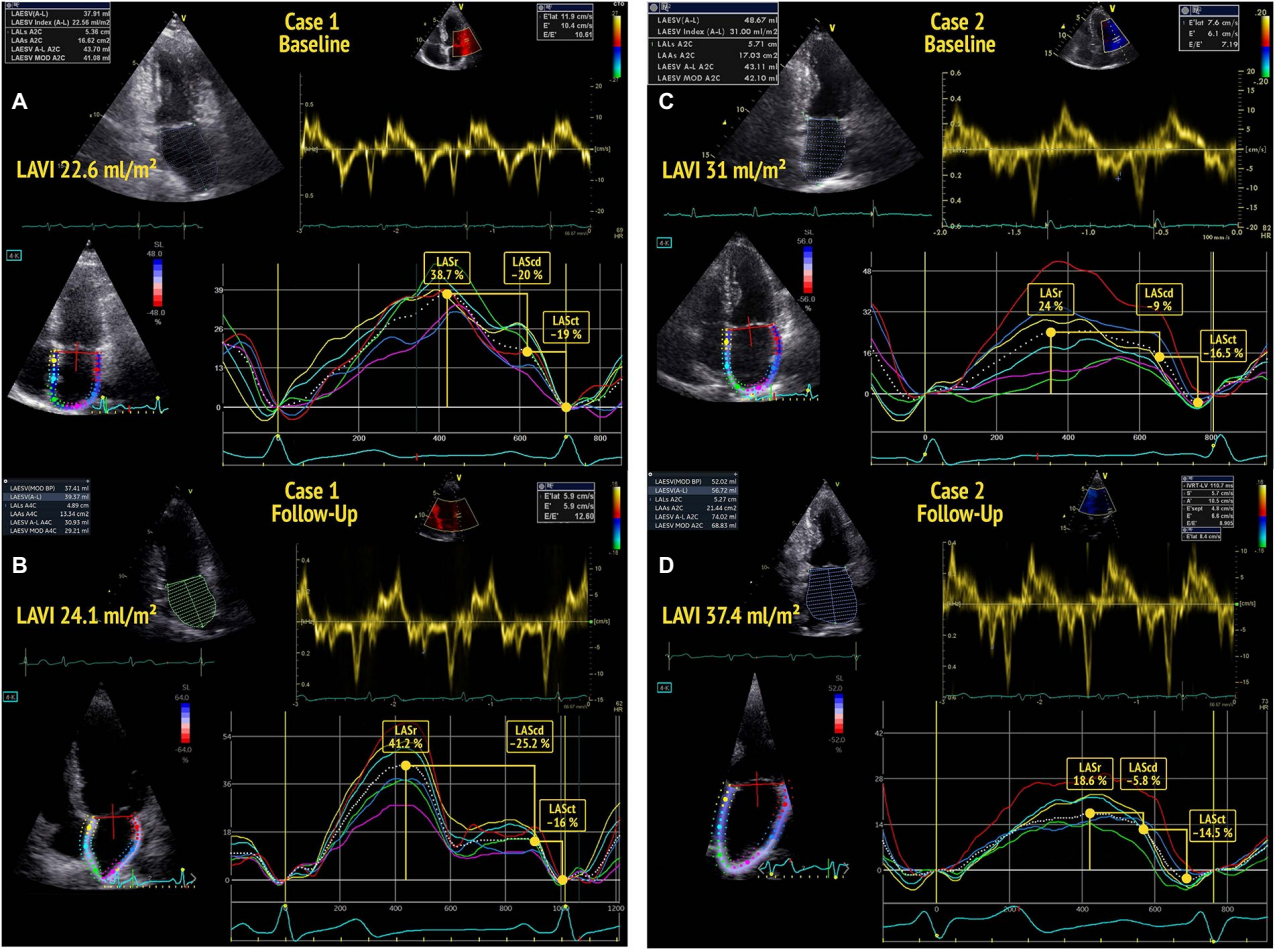
Baseline echocardiography of two participants **(A,C)** showing normal left atrial volume index (LAVI) (<34 ml/m^2^) and normal diastolic function according to the 2016 ASE guidelines. The participant in A shows a left atrial reservoir strain (LASr) >34% at baseline and no deterioration of diastolic function by the time of follow-up **(B)**. The participant in C presents with a low baseline LASr and shows a decline of diastolic function (DD0 to DD1) by the time of follow-up echocardiography **(D)**.

2D STE strain studies were analyzed offline using the EchoPAC v203 software (GE Healthcare). Global peak systolic longitudinal LV strain (LV GLS) was determined from apical 4-chamber, 2-chamber, and long-axis views (17 segment LV model). Phasic LAS was assessed as proposed by the recent EACVI recommendations (12) from an LA focused apical 4-chamber-view, avoiding foreshortening. Three cardiac cycles were recorded for each view and stored for offline analysis. Gain, depth, and frame rate (60–80 frames/s) were optimized for image acquisition. The region of interest was placed on the atrial walls, distributing the interatrial septum and atrial free wall into six segments. LAS was analyzed QRS-triggered. LASr was identified from the plotted average strain curve as the maximum amplitude during ventricular systole. LA conduit strain (during passive LV filling; LAScd) and LA contraction strain (during peak atrial contraction; LASct) were calculated from the generated strain curve as previously described (4, 12, 22) (Figure 1).

### Classification of DD

Diastolic function was categorized by an experienced cardiologist blinded for the clinical data in accordance with the recent ASE/EACVI recommendations on LV diastolic function (6). The following parameters were used for classification: (a) septal or lateral e′ velocity of <7 cm/s or <10 cm/s; or average e′ <9 cm/s; (b) E/e′ >14; (c) RV-RA pressure difference >31 mmHg (corresponding to a TR velocity >2.8 m/s); or (d) LAVI >34 ml/m^2^. Study participants who fulfilled 50% of these diagnostic criteria were graded to have signs of impaired diastolic function (DD1) while subjects meeting >50% of these criteria were assigned to the DD2 group (overt DD) (6). All others fell in the group without DD (DD0).

Applying these criteria to data of both measurement occasions let us define a progression variable that indicates whether the DD status of the woman deteriorated (DD0 to DD1 or DD1 to DD2; labeled “progression”) or remained the same (stable in DD0 or DD1; labeled “no progression”).

### Statistical analysis

Descriptive statistics for baseline and follow-up measures were computed for the full sample and subgroups based on a combination of baseline DD status and DD progression.

To learn more about which baseline measures are associated with a worsening of diastolic function, we performed subsequent backward stepwise logistic regression analyses. In a first step, we build a reference model that included age, BMI, arterial hypertension, diabetes, renal function, average e′, E/e′ and LAVI as predictors and “progression” as the outcome. Starting with this full model, we then deleted terms with a backwards algorithm based on the AIC. The only remaining predictors were age, LASr, and hypertension. The model we report contains in addition e′ and LAVI to demonstrate the relative performance of the competing echo measures. In an extended model, a LAS measure was then added. Because the two LAS indicators – LASr and LAScd – are highly correlated (−0.85), we decided to include only LASr or LAScd, respectively, as predictor. All predictors were centered at their mean value. Models were compared using a likelihood ratio test, information criteria, and Pseudo- *R*^2^ (Nagelkerke) measures. A *p*-value <0.05 was considered statistically significant. In addition, receiver operating characteristic (ROC) curve analyses were performed to assess the diagnostic value of phasic LASr, LAScd, LAVI, and e′ velocity. All analyses were done in R (R Core Team, 2021) with usage of the additional package pROC (23, 24).

## Results

### Study population

Mean follow-up time was 6.8 years (353.1 weeks; interquartile range 343.1–361.1 weeks). Of 449 study subjects reinvited in written form for follow-up examinations, 332 gave written consent (response rate 73.9%). Of these 332 participants, two visits were canceled due to unexpected other medical procedures, nine patients were lost to follow-up, and 63 scheduled follow-up examinations were canceled due to restrictions in relation to the SARS-CoV-2-pandemic situation. Of the 258 participants who were included in the study, two were excluded from analysis due to the development of moderate to severe mitral regurgitation and atrial fibrillation, five because of poor acoustic window, and two due to reduced speckle tracking quality of the LA wall resulting in a total follow-up sample size of 249 women.

### Subgroups

Demographic and clinical characteristics of the total sample and subgroups depending on initial status and progression are shown in Table 1. Of the 249 participants, 29 showed DD1 at baseline. Of those 29 women, six (20.7%) had progressed to DD2 by the time of the follow-up examination. Of the 220 women with initially normal diastolic function, 34 (15.5%) had developed DD1 or DD2. The women whose diastolic function had deteriorated were older, had a higher BMI, showed higher levels of natriuretic peptides, and suffered more often from cardiovascular risk factors or diseases (Table 1).

### Descriptive echocardiography and reproducibility of measurements

**Figure 2:**
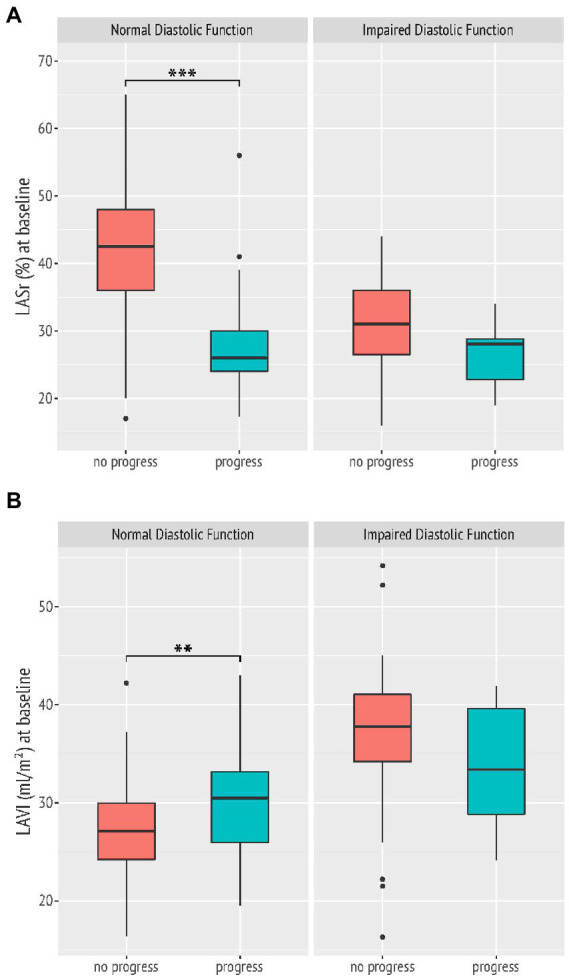
Left atrial reservoir strain (LASr) is significantly reduced **(A)**, while left atrial volume index (LAVI) is markedly higher, but still below the recommended cut-off value of 34 ml/m^2^ **(B)** in study participants with normal diastolic function at baseline who then showed a deterioration of diastolic function (progress) by follow-up. ***p < 0.001; **p = 0.004.

Descriptive statistics for echocardiographic measures and myocardial mechanics at baseline and follow-up can be found in Table 2. Participants who developed DD over time showed a reduced LASr and LAScd value at baseline compared to women whose diastolic function remained in the normal range, while the average LAVI was significantly higher but – according to the currently recommended cut off value of 34 ml/m^2^ – still in the normal range (Table 2; Figure 2; Supplementary Figure 1). Echocardiographic parameters including myocardial mechanics at baseline and at the time of follow-up are shown in Table 2. With regard to follow-up data, previously healthy women who developed DD over time showed a significantly lower LASr and LAScd and a markedly increased LAVI by the time of follow-up (Table 2). Differences in clinical and echocardiographic characteristics between groups were tested using analysis of variance for continuous variables and the Fisher’s exact test for categorial variables. Intra- and interobserver variability of phasic LAS measurements are published and have been shown to be very low (4).

**Table 2:** Echocardiographic characteristics and myocardial mechanics.

	Total sample	Subgroups				*p*-value
		DD0 at baseline		DD1 at baseline		
		No progress	Progress	No progress	Progress	
Group size	249	186	34	23	6	
**Baseline**
Echocardiography					
LVEF, %	60.5 ± 5.7	61 ± 6	59.7 ± 7.0	57.8 ± 5.2	61.0 ± 7.9	0.071
IVSD, mm	9.8 ± 1.8	9 ± 1	11 ± 1.5	11 ± 2	13.2 ± 1.7	<0.001
PWD, mm	9.4 ± 1.5	9 ± 1	10 ± 1.5	10 ± 1	11.8 ± 1.5	<0.001
LVEDD, mm	45.2 ± 4.7	45.2 ± 4.7	45.0 ± 4.9	45.7 ± 4.1	43.2 ± 4.1	0.69
E, cm/s	0.7 ± 0.2	0.8 ± 0.2	0.7 ± 0.2	0.7 ± 0.2	0.7 ± 0.2	<0.001
A, cm/s	0.6 ± 0.2	0.6 ± 0.2	0.7 ± 0.2	0.7 ± 0.2	0.8 ± 0.1	<0.001
E/A	1.3 ± 0.6	1.4 ± 0.6	1.0 ± 0.4	0.9 ± 0.3	0.9 ± 0.3	<0.001
DT, ms	211 ± 47	207 ± 43	225 ± 55	214 ± 57	229 ± 59	0.073
IVRT, ms	78 ± 23	73 ± 20	92 ± 21	94 ± 31	91 ± 28	<0.001
e′ average, cm/s	11.3 ± 3.4	12.4 ± 3.1	8.7 ± 2.0	7.6 ± 1.3	6.0 ± 1.2	<0.001
E/e′	7.0 ± 2.1	6.5 ± 1.7	7.7 ± 1.5	9.1 ± 2.4	11.6 ± 1.6	<0.001
LAVI, ml/m^2^	28.5 ± 6.0	27.0 ± 4.5	30.0 ± 5.3	36.8 ± 8.9	33.6 ± 7.1	<0.001
∆RV/RA pressure, mmHg	21.8 ± 5.8	20.5 ± 5.4	22.7 ± 5.7	25.4 ± 5.8	28.5 ± 6.4	0.008
TAPSE, mm	24.0 ± 4.1	24.1 ± 4.3	23.6 ± 3.9	24.3 ± 3.2	21.5 ± 3.6	0.48
Myocardial mechanics						
LASr (%)	38.6 ± 9.9	41.9 ± 8.5	28.0 ± 7.0	30.6 ± 7.1	26.5 ± 5.6	<0.001
LAScd (%)	−22.4 ± 9.7	−25.4 ± 9.1	−13.2 ± 5.1	−14.9 ± 5.7	−13.0 ± 1.6	<0.001
LASct (%)	−17.9 ± 5.5	−18.1 ± 5.4	−17.6 ± 6.3	−17.7 ± 5.8	−15.9 ± 5.3	0.52
LV GLS (%)	−20.6 ± 2.5	−21.2 ± 2.1	−19.4 ± 2.8	−18.5 ± 2.2	−17. 6 ± 2.9	<0.001
**Follow-up**						
Echocardiography						
LVEF, %	60.4 ± 3.7	60.6 ± 3.6	60.4 ± 4.1	58.6 ± 3.6	60.7 ± 5.5	0.092
IVSD, mm	10.2 ± 1.8	10 ± 2	11 ± 1.6	12 ± 2	14 ± 2.0	<0.001
PWD, mm	9.6 ± 1.6	9 ± 1	11 ± 1.4	11 ± 1	12 ± 1.9	<0.001
LVEDD, mm	43.9 ± 4.1	43.9 ± 4.1	44.0 ± 4.4	44.4 ± 4.7	43.6 ± 4.4	0.94
E, cm/s	0.7 ± 0.2	0.7 ± 0.2	0.6 ± 0.1	0.6 ± 0.1	0.7 ± 0.1	0.001
A, cm/s	0.6 ± 0.2	0.6 ± 0.2	0.7 ± 0.2	0.8 ± 0.2	0.7 ± 0.1	<0.001
E/A	1.2 ± 0.5	1.3 ± 0.5	0.9 ± 0.3	0.8 ± 0.3	1.1 ± 0.4	<0.001
DT, ms	194 ± 45	190 ± 43	207 ± 49	205 ± 50	231 ± 43	0.004
IVRT, ms	92 ± 27	86 ± 24	104 ± 17.9	115 ± 32	133 ± 35	<0.001
e′ average, cm/s	9.6 ± 3.2	10.7 ± 2.9	6.9 ± 1.0	6.3 ± 1.1	4.8 ± 2.0	<0.001
E/e′	7.8 ± 2.5	7.0 ± 1.7	9.6 ± 2.5	9.5 ± 1.8	15.9 ± 4.1	<0.001
LAVI, ml/m^2^	31.0 ± 6.6	28.4 ± 4.0	39.0 ± 5.8	38.3 ± 8.6	38.5 ± 2.8	<0.001
∆RV/RA pressure, mmHg	23.4 ± 6.8	19.1 ± 4.5	23.1 ± 6.5	22.1 ± 3.8	32.7 ± 4.9	<0.001
TAPSE, mm	23.5 ± 6.8	23.9 ± 7.6	21.9 ± 3.1	22.8 ± 2.9	22.3 ± 3.4	0.40
Myocardial mechanics						
LASr (%)	34.0 ± 9.5	37.1 ± 8.5	24.5 ± 5.4	25.5 ± 5.5	21.6 ± 3.9	<0.001
LAScd (%)	−18.4 ± 8.1	−21.0 ± 7.6	−10.7 ± 4.0	−10.5 ± 3.0	−9.1 ± 2.6	<0.001
LASct (%)	−16.0 ± 5.3	−16.3 ± 5.6	−14.6 ± 3.5	−16.3 ± 5.6	−13.4 ± 4.3	0.29
LV GLS (%)	−20.6 ± 3.7	−21.0 ± 4.0	−20.2 ± 2.2	−18.8 ± 2.3	−18.7 ± 1.4	0.025

LVEF, left ventricular ejection fraction; IVSD, interventricular septal diameter; PWD, posterior wall diameter; LVEDD, left ventricular enddiastolic diameter; E and A, mitral peak E- and A-wave velocity; DT, deceleration time; IVRT, isovolumetric relaxation time; e′, pulsed-wave TDI-derived mitral annular early diastolic velocity; LAVI, left atrial volume index; ∆RV/RA pressure, systolic right atrial/right ventricular pressure difference; TAPSE, tricuspid annular plane systolic excursion; LASr, LAScd, and LASct, left atrial reservoir, conduit, and contraction strain; LV GLS, global longitudinal peak systolic left ventricular strain.

### Predictors of DD development

#### ROC analysis

**Figure 3:**
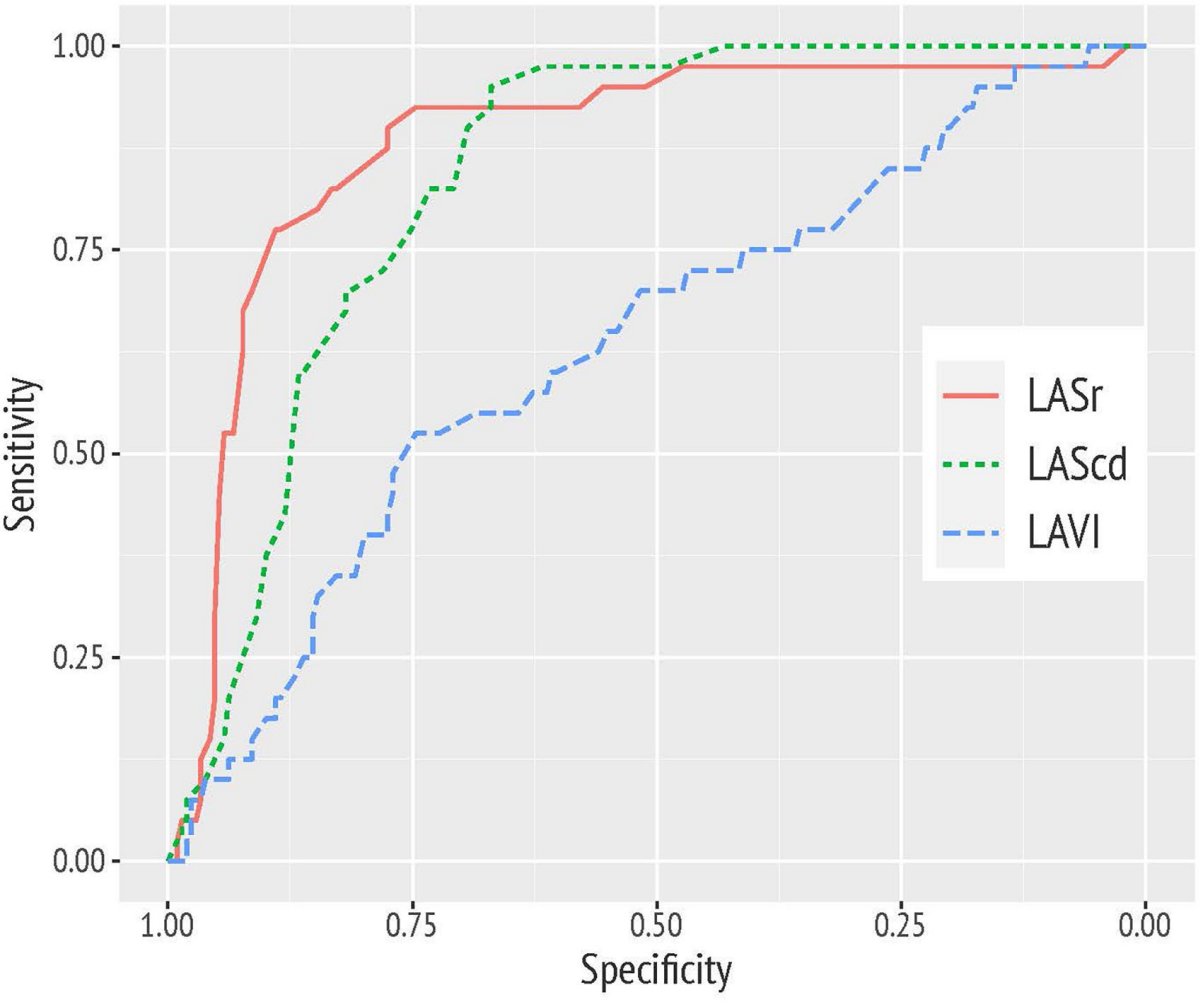
Receiver operating characteristic (ROC) curve analysis demonstrating the superior diagnostic value of left atrial reservoir strain (LASr) and conduit strain (LAScd) compared to left atrial volume index (LAVI) to discriminate study participants with a worsening of diastolic function over time.

With an AUC of 0.88 (95% CI 0.82–0.94) and an AUC of 0.84 (95% CI 0.79–0.89), LASr and LAScd values showed a high discriminative power in predicting a decline of diastolic function over time. These LA function parameters performed significantly better than the echocardiographic standard parameter LAVI (AUC 0.63; 95% CI 0.54–0.73; see Figure 3). Average e′ held a good discriminative value, as well (AUC of 0.80 with 95% CI 0.74–0.86; not graphically represented).

For a cut point of <34%, LASr was associated with a sensitivity of 90% and a specificity of 78% in predicting the worsening of diastolic function. For LAScd, respective sensitivity and specificity were 83 and 73% for a cut off value >−17.5%.

### Descriptive risk of diastolic function worsening

31 of 34 (91.2%) of the previously healthy women who – according to recent ASE/EACVI-recommendations – showed a decrease of diastolic function by follow-up showed a reduced baseline LASr according to our ROC analysis (LASr <34%). In contrast, only 6 of the 34 women (17.6%) featured a LAVI >34 ml/m^2^, 17 (50.0%) showed an average e′ <9.0 cm/s (1). Vice versa, of the 63 participants who were classified to have DD0 and a LASr <34% at the time of baseline examinations, 31 then developed DD at follow-up (49%) compared to only 2% (3 of 157) participants with a baseline LASr ≥34%. Estimation of PA pressure and assessment of E/e′ was not helpful in discriminating patients with development of DD since PA pressure was not assessable in 157 patients, and baseline E/e′ was <14 in 248 participants.

### Logistic regression analyses

When comparing the reference model with established measures and the extended model, including LASr improved the model as indicated by a significant Likelihood Ratio Test (*χ*^2^(1) = 28.9, *p* < 0.001), a lower AIC (181.1 vs. 154.2), and a higher Nagelkerke Index (0.35 vs. 0.49).

**Figure 4:**
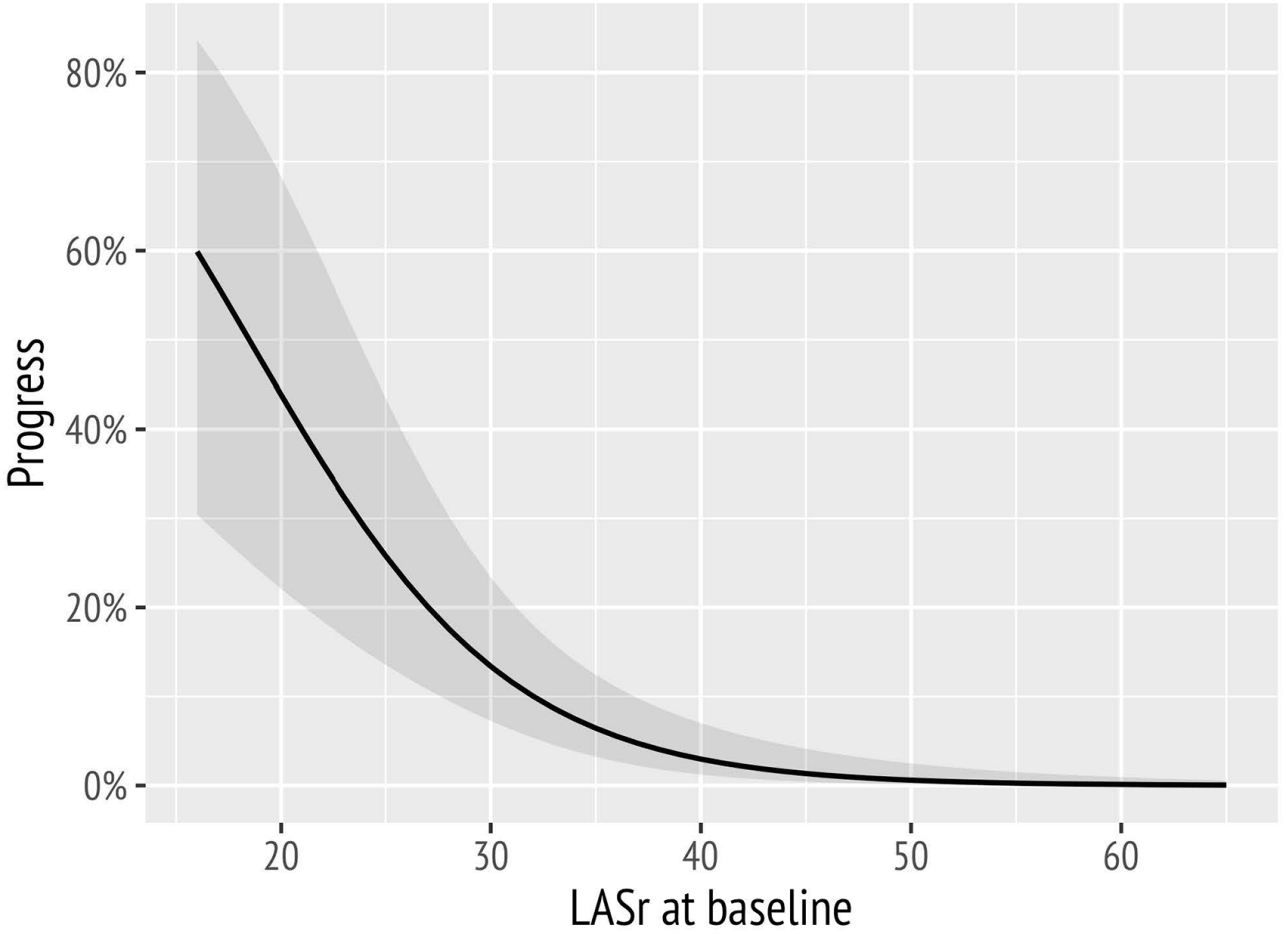
Probability of diastolic function worsening over time markedly increased with decreasing baseline left atrial reservoir strain (LASr) values.

The results of the extended model are presented in Table 3. In this model, LASr (Table 3) remained a significant predictor of a decline of diastolic function when correcting for age, hypertension, e′ average, and LAVI. LAScd was significantly associated with a worsening of diastolic function, as well (Supplementary Table 1); however, including LAScd in addition to LASr in the analysis did not improve the extended model (data not shown). In addition, the conditional effects of LASr are illustrated in Figure 4.

**Table 3:** Predictors of diastolic function worsening over time; multivariate logistic regression analysis.

	*B*	SE	*z*	*p*-value	BETA	LL	OR	UL
Constant	−3.20	0.42	−7.60	<0.001	−3.20	0.02	0.04	0.09
Age	0.04	0.03	1.57	0.12	0.57	1.00	1.04	1.10
Hypertension	1.12	0.47	2.37	0.018	1.12	1.22	3.08	7.94
e′ average	−0.05	0.11	−0.50	0.62	−0.18	0.77	0.95	1.17
LAVI	−0.02	0.03	−0.52	0.60	−0.11	0.92	0.98	1.05
LASr	−0.16	0.03	−4.80	<0.001	−1.59	0.80	0.85	0.91

SE, standard error; *z*, *z*-value, LL und UL, lower and upper limits of 95% confidence interval; OR, odds ratio; e′ average, pulsed-wave TDI-derived mitral annular early diastolic velocity; E, mitral peak E-wave velocity; LAVI, left atrial volume index; LASr, LA reservoir strain.

## Discussion

This longitudinal study demonstrates that phasic LAS entails predictive value in evolving DD. Phasic LAS analysis was associated with a higher diagnostic accuracy in predicting diastolic function worsening than LAVI and other echocardiographic parameters in study participants with a normal diastolic function at baseline. Furthermore, subjects with baseline LASr <34% demonstrated a significantly increased risk of developing impaired diastolic function over time. The analysis of LA mechanics may accordingly be not only useful as a diagnostic but also as a predictive tool in the echocardiographic assessment of diastolic function.

Current guidelines (6) still recommend to assess LAVI due to its large body of evidence in cardiovascular risk stratification (25). Although not yet included in the ASE/EACVI recommendations, the analysis of LA function might significantly improve the predictive impact of the echocardiographic assessment in diverse clinical conditions. Our data additionally suggest that alterations of phasic LAS, and not of LAVI, may be useful to identify “healthy” patients at risk for future DD development.

Strain alterations of all three phases of the LA cycle have been widely described in DD and there is reliable evidence that LAS may worsen even in absence of LA volume enlargement (1, 3, 9). LASr hence constitutes an accurate, feasible and robust parameter in the evaluation of DD and has recently been proposed as a sensitive and specific parameter to be included in the diagnostic algorithm of DD (26). This may lead to an improvement in terms of detection and classification of the severity of DD, especially in situations where classification cannot be unequivocally achieved by current guideline algorithms (“gray zone” or indeterminate range) (27–29). As previously shown in the BEFRI trial (4), all three LAS components exhibit specific alterations in different DD stages. We were able to confirm these findings in the present follow-up study. In line with these findings, Morris et al. (9) demonstrated higher sensitivity of LAS analyses compared with LAVI in detecting DD, as well. In their study, 62% of patients with DD presented a LASr <23%, while only 34% showed a LAVI >34 ml/m^2^. Furthermore, the addition of LASr to the standard algorithms improved the detection of DD by 10% (9). The fact that LA mechanics are essential to preserve LV function (30) and the close relationship to symptom development (9, 31, 32) brings LAS assessment in the focus of early detection methods of DD (26). Our study further demonstrates that decreased phasic LAS may be of predictive value with respect to the worsening of diastolic function.

There is increasing evidence that analysis of LA function may play a key role as a prognostic marker in patients with advanced DD. For example, the TOPCAT trial demonstrated that a reduction of LASr was associated with a higher risk of HF hospitalization and cardiovascular death (18). In the previously mentioned study by Morris et al. (9), a LASr <23% was linked to worse NYHA class and higher risk of hospitalization for HF. Similarly, LASr was found to be an independent predictor of death and cardiovascular hospitalization in 308 HFpEF patients (32).

To our knowledge, the present study is the first to prospectively investigate the predictive value of phasic LAS alterations with respect to a decline of diastolic function. LASr and LAScd appeared superior to predict worsening of diastolic function over time than LAVI and other traditional DD parameters. Consequently, phasic LAS analysis may contribute to an improved diagnostic work up and risk stratification in patients with normal diastolic function but at risk for future diastolic function worsening.

## Limitations

Diastolic function was strictly graded according to the current ASE recommendations on diastolic function with all its inherent limitations, i.e., providing a good specificity with regard to overt clinical DD while potentially missing a gradual decrease of diastolic function. Following rigorously the criteria of the current DD grading scheme, phasic LAS analysis proved superior to LAVI in predicting a decline of diastolic function in patients without DD; this diagnostic strength suggests a potential role in the management of patients at risk in future. Age-related cut offs in DD have recently been defined and found to be of prognostic significance in patients with mild DD (33). As another limitation, our analyses were not stratified by age. However, in multivariate analyses controlling for age among other factors, LASr and LAScd remained significantly associated with a worsening of diastolic function over time. Due to the study design of the BEFRI study, only women were included in the analyses. Although previous reports have found no differences between LAS in men and women (13, 34) the predictive value has yet to be demonstrated in studies of men. Due to restrictions of the pandemic SARS-CoV-2 situation, more than 60 patients were unpredictably lost to follow up. However, the results of the ROC and logistic regression analyses showed a strong additive predictive utility of phasic LAS analysis on the course of DD that warrant further studies, preferably including both, men and women. In addition, the clinical impact of our data needs to be investigated by larger future trials which yield sufficient power to demonstrate associations between LAS alterations and the development of HF signs and symptoms, preferably using a LA-dedicated strain tracking software that was not yet available for clinical use at the time of baseline investigations.

## Conclusion

Our data demonstrated a high discriminative value of phasic LAS analysis to predict a decline of diastolic function in study participants with normal baseline diastolic function in a longitudinal study design. The integration of phasic LAS analysis into the current echocardiographic algorithm to assess diastolic function may be useful for predicting the deterioration of LV diastolic function and therefore for identifying patients at risk for a future development of DD.

## Data availability statement

The datasets presented in this article are not readily available because of continued data analyses. Requests to access the datasets should be directed to anna.brand@charite.de.

## Ethics statement

The studies involving human participants were reviewed and approved by Charité-Universitätsmedizin Berlin (EA/2085/19). The patients/participants provided their written informed consent to participate in this study.

## Author contributions

AB: funding of study, conception and design, data collection, supervision of study, analysis and interpretation, writing the manuscript, critical revision of manuscript, and funding. ER: data collection, analysis and interpretation, writing the manuscript. AW and DB-W: data collection, analysis and interpretation, critical revision of manuscript. US: funding of study, conception and design, critical revision of manuscript. CC: statistical analysis, analysis and interpretation, critical revision of manuscript. SB: analysis and interpretation, critical revision of manuscript. IM: data collection, analysis and interpretation, critical revision of manuscript. HD, KS, VR-Z, UL, and FK: conception and design, analysis and interpretation, critical revision of manuscript. VS: conception and design, analysis and interpretation, writing the manuscript, critical revision of manuscript. All authors contributed to the article and approved the submitted version.

## Funding

This study was supported by the DZHK (German Center for Cardiovascular Research) and by the BMBF (German Ministry of Education and Research). Grant number 81Z0100211.

## Conflict of interest

The authors declare that the research was conducted in the absence of any commercial or financial relationships that could be construed as a potential conflict of interest.

## Publisher’s note

All claims expressed in this article are solely those of the authors and do not necessarily represent those of their affiliated organizations, or those of the publisher, the editors and the reviewers. Any product that may be evaluated in this article, or claim that may be made by its manufacturer, is not guaranteed or endorsed by the publisher.
