# Supplementary figures and images for: Phasic left atrial strain to predict worsening of diastolic function: Results from the prospective Berlin Female Risk Evaluation follow-up trial
Source: Front Cardiovasc Med. 2023 Feb 20;10:1070450. doi: 10.3389/fcvm.2023.1070450 (PMC9986257; doi:10.3389/fcvm.2023.1070450)

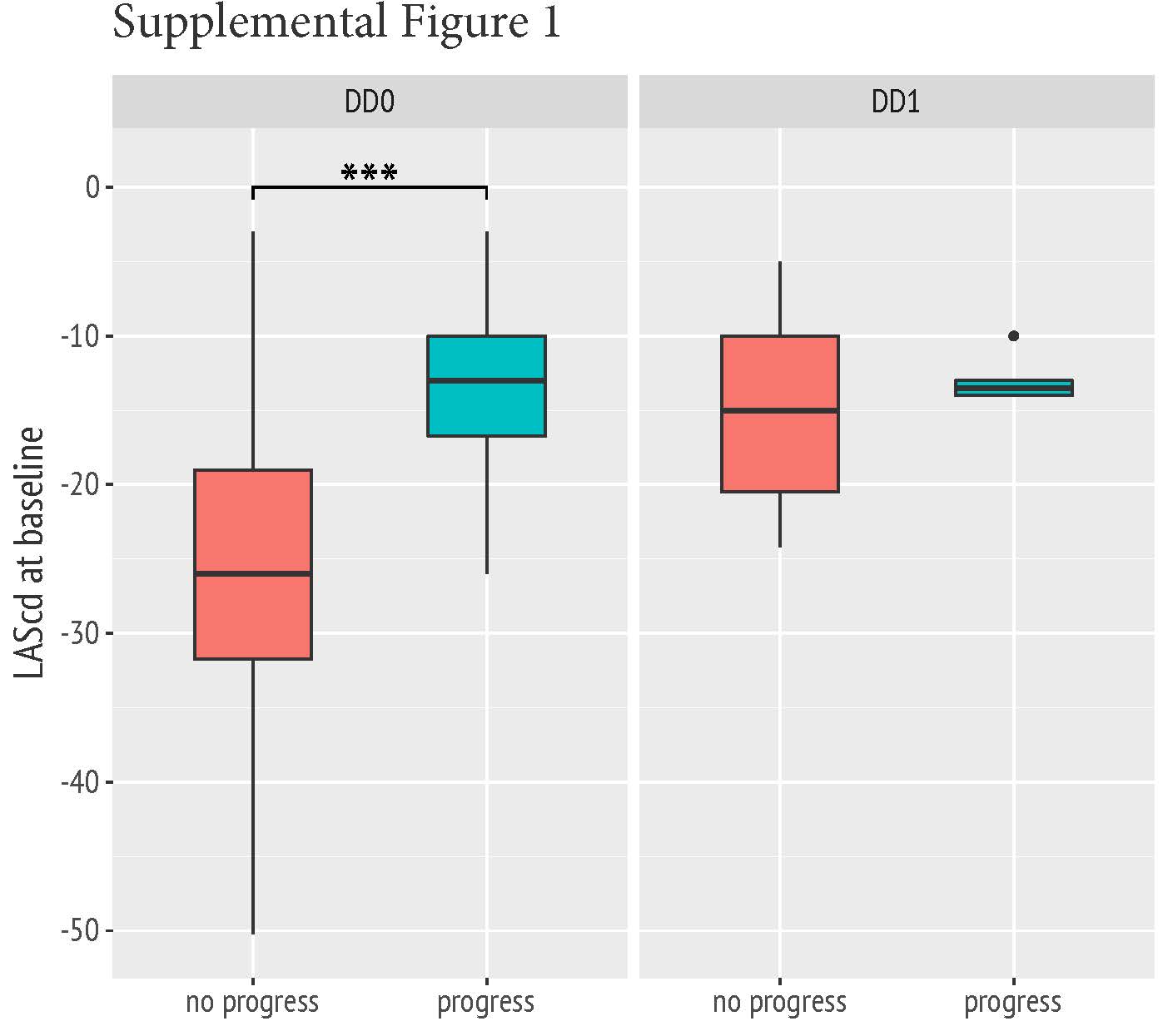

Supplement: Supplementary file 1 [file Image_1.JPEG]
