# Supplementary material for: Phasic left atrial strain to predict worsening of diastolic function: Results from the prospective Berlin Female Risk Evaluation follow-up trial
Source: Front Cardiovasc Med. 2023 Feb 20;10:1070450. doi: 10.3389/fcvm.2023.1070450 (PMC9986257; doi:10.3389/fcvm.2023.1070450)
Supplement: Supplementary file 2 [file Table_3.DOCX]

**Supplement**

**Supplementary Figure 1:** LA conduit strain is significantly reduced in study participants with normal diastolic function at baseline who then showed a deterioration of diastolic function (progress) by follow-up. ***p<0.001.

**Supplementary Table 1: Predictors of diastolic function worsening over time; multivariate logistic regression analysis**

|  | B | SE | z | p-value | LL | OR | UL |
| --- | --- | --- | --- | --- | --- | --- | --- |
| (Intercept) | -2.588 | 0.352 | -7.344 | 0.000 | 0.035 | 0.075 | 0.140 |
| Alter | 0.626 | 0.342 | 1.828 | 0.067 | 0.965 | 1.870 | 3.731 |
| BMI | 0.037 | 0.235 | 0.158 | 0.874 | 0.643 | 1.038 | 1.627 |
| e’ average | -0.254 | 0.421 | -0.603 | 0.547 | 0.332 | 0.776 | 1.749 |
| EE | 0.046 | 0.257 | 0.181 | 0.857 | 0.626 | 1.047 | 1.730 |
| LAVI | 0.045 | 0.181 | 0.249 | 0.803 | 0.731 | 1.046 | 1.497 |
| LAScd | 1.253 | 0.342 | 3.663 | <0.001 | 1.835 | 3.502 | 7.098 |
| SE, standard error; z, z-value, LL und UL, lower and upper limits of 95% confidence interval; OR, odds ratio; e’ average, pulsed-wave TDI-derived mitral annular early diastolic velocity; E, mitral peak E-wave velocity; LAVI, left atrial volume index; LAScd, LA conduit strain | | | | | | | |
